# Organic Electrochemical Transistors for Real-Time Detection of Immune System Early Activation during Atezolizumab-Bevacizumab Treatment in Hepatocellular Carcinoma

**DOI:** 10.1021/acsami.6c03306

**Published:** 2026-04-29

**Authors:** Francesco Decataldo, Andrea Arleo, Annapaola Montagner, Giorgio Cortelli, Cristina S. Cioroianu, Fabio Piscaglia, Beatrice Fraboni, Laura Gramantieri, Catia Giovannini

**Affiliations:** ∥ Department of Medical and Surgical Sciences, 9296 Alma Mater Studiorum-University of Bologna, 40138 Bologna, Italy; ‡ Department of Physics and Astronomy, Alma Mater Studiorum-University of Bologna, 40127 Bologna, Italy; § Division of Internal Medicine, Hepatobiliary and Immunoallergic Diseases, IRCCS Azienda Ospedaliero-Universitaria of Bologna, 40138 Bologna, Italy

**Keywords:** HCC, Immunotherapy, PBMC, Cocultures, Organic Electrochemical Transistors, Real-Time Cell Health Monitoring

## Abstract

Poly­(3,4-ethylenedioxythiophene):polystyrenesulfonate (PEDOT:PSS)-based organic electrochemical transistors (OECTs) have been demonstrated as versatile biosensors in recent years. Owing to the biocompatibility and chemical stability of the organic mixed ionic and electronic conductor in cell media, they allow real-time, in vitro electrical monitoring of cell lines, providing quantitative outcomes of their health status. Here, we propose and validate a sensitive OECT device for direct in vitro monitoring of peripheral blood mononuclear cell (PBMC) activity in a coculture assay. PBMCs from patients with advanced hepatocellular carcinoma (HCC) responding and nonresponding to atezolizumab (anti-PD-L1) and bevacizumab (anti-VEGF) are cocultured with Huh7 and SNU449 HCC cell lines directly grown onto OECTs. A strong correlation between the OECT electrical response, standard cell growth, death assays, and IL6 levels is observed, which is confirmed for cocultures involving PBMCs from healthy controls and patients treated with immunosuppressive drugs (namely, positive and negative controls, respectively). Our low-cost and scalable device has the potential to detect PBMC activation induced by atezolizumab–bevacizumab in the very early stages of HCC treatment, allowing for nonresponders’ inclusion in alternative treatments. This approach might be of great interest to several human cancers treated with immune checkpoint inhibitors (ICIs), providing sensitive, automated, and noninvasive tools to complement clinical practice.

## Graphical abstract

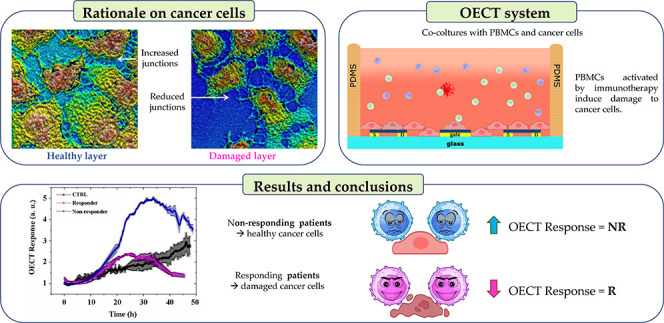

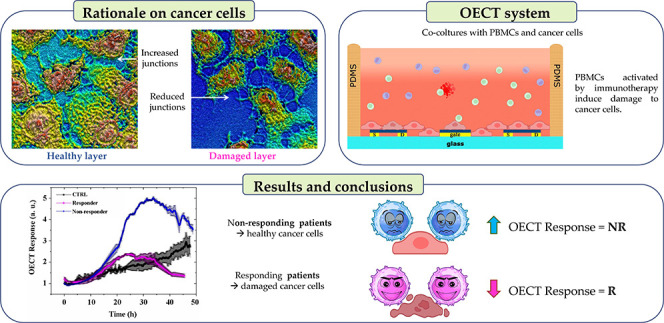

## Introduction

Organic electrochemical transistors (OECTs) are three terminal devices employing semiconducting polymers as channel and/or gate material.^1^ Among the several polymers that have been used for OECT fabrication, p-type poly­(3,4-ethylenedioxythiophene):polystyrenesulfonate (PEDOT:PSS) represents one of the most employed due to its chemical stability, easy processability, and low cost. Research on PEDOT:PSS OECT biosensors has strongly increased in recent years, benefiting from their in situ signal amplification, high sensitivity, and low power consumption as well as use in a wide spectrum of biological and bioelectronic applications. Besides their employment for physiological recordings/stimulation^2–4^ and as neuromorphic devices,^5,6^ OECTs have been mainly applied for chemical (e.g., analytes, ions, and DNA)^7,8^ or impedance biosensing.^9–11^ When employed as impedance sensors, the polymer biocompatibility and its stability in aqueous environment allow for direct interfacing of cells to OECT active areas. Cell seeding and growth directly onto the polymeric thin film enable intimate cell–surface contact and long-term, continuous in vitro experiments, which are crucial for investigating physiological processes.^12–14^ Recently, we demonstrated that OECTs are versatile tools for assessing cytotoxicity, neutralizing antibody response toward viruses, and evaluating viral effect, overcoming optical analysis limitations.^15–17^

Herein, we explored whether an OECT can be used as an advanced in vitro model to study the interactions between peripheral blood mononuclear cells (PBMCs) and cancer cells, namely, coculture, to verify the activation of PBMCs isolated from blood of hepatocellular carcinoma (HCC) patients during immunotherapy. Indeed, immune cells, including lymphocytes and monocytes, are key players in response to immune checkpoint inhibitors (ICIs) across a wide range of cancers including HCC, the primary malignant tumor of the liver, whose mortality and incidence are constantly growing.^18^ Immunotherapy with atezolizumab (anti-PDL-1) and bevacizumab (anti-VEGF) has strongly improved the survival of patients with advanced HCC; however, approximately 30% of patients do not respond to treatment, and a progression after an initial response occurs at 12–15 months in a relevant percentage of cases.^19^ According to clinical practice, the response to treatment is established every 3 months by imaging. Besides possible technical difficulties in the assessment of response, such in the case of pseudoprogression, upfront biomarkers might help personalize therapeutic approaches and follow-up. To this aim, cocultures with tumor cells and PBMCs isolated from patients before treatment and 3 weeks after drug infusion are promising readouts of immune response activation. The technologies currently available for cocultures range from transwell plates to microfluidics, but the acquisition and interpretation of results are still major limitations. The problem is compounded by the lack of rapid, simple, and inexpensive assays for cocultures that allow such experiments to be carried out more routinely in clinical practice. Preliminary experiments and protocols have been done using multi-electrode arrays for coculture monitoring,^20,21^ but this configuration lacks the inherent transistor amplification and often hinders a complete and reliable optical image acquisition due to nontransparent, metallic electrodes. By using OECT technology, we established a simple, fast, and reliable coculture investigation model of HCC cell lines and PBMC (isolated from healthy and HCC patients) to simulate the interaction between immune tricky and tumor cells, avoiding protocols or architecture (i.e., transwell). Interestingly, the cell layer was monitored in real-time with fast temporal resolution, thus reconstructing the whole coculture interaction while still allowing optical evaluation (owing to the transparency of the 100 nm thin PEDOT:PSS active layers) and end-point assays (if needed). The device switch-off time response upon a positive voltage pulse on the gate (i.e., the time required for current switching from the “on” state to the “off” one) was directly correlated to the tissue integrity and health of the cell layer, thus specifically associated with the PBMC effect against the cell line. This approach was also able to correctly identify the response to treatment of patients missed by other tests,^22^ suggesting the usefulness and reliability of OECTs to set up cocultures as noninvasive predictive tools for evaluating the response to atezolizumab–bevacizumab in the very early stages of HCC treatment.

## Experimental Section

### Cell Culture

SNU449 and HepG2 cell lines were purchased from the American Type Culture Collection (ATCC, Rockville, MD, USA), and Huh7 cells were from Prof. Alberti’s laboratory, University of Padua (Padua, Italy). SNU449 and Huh7 were cultured in RPMI, while HepG2 cells were cultured in MEM. Media were supplemented with 10% FBS and 1% P/S and maintained in an incubator with a 95% humidified atmosphere containing 5% CO_2_ at 37 °C. Cells were routinely tested for mycoplasma contamination.

### Patients and Healthy Donors

Patients with primary HCC not amenable for curative or locoregional treatments and without active HBV or HCV infection were enrolled in the study and referred to S. Orsola-Malpighi University Hospital of Bologna. All patients were treated by atezolizumab (1200 mg as an intravenous infusion) in association with bevacizumab (15 mg/kg intravenous infusion) on the same day according to a 3-week infusion schedule. Patients’ management and assessment of response to treatment were performed according to clinical practice.^23^ Three patients undergoing a suppressive regimen after orthotopic liver transplantation (OLT) and 3 healthy donors were also included in the study approved by the local ethics committee (Comitato Etico Area Vasta Emilia Centro—AVEC) on 6 June 6, 2021 (approval number 528/2021/Sper/AOUBo). The study was conducted in accordance with the 1964 Helsinki declaration and its later amendments. Written informed consent was obtained from all of the individual participants included in the study.

### Peripheral Blood Mononuclear Cell (PBMC) Isolation

Peripheral blood was collected on a Ficoll gradient vacutainer (Becton, Dickinson and Company, Franklin Lakes NJ, USA) for PBMC separation as previously described.^22^ Once separated, a fraction of PBMC was diluted in RPMI, counted, and used to establish cocultures with HCC cell lines.

### OECT Device Fabrication

Glass substrates (25 × 25 mm^2^) were cleaned by sonication in acetone/isopropanol/distilled water baths. Afterward, substrates were dehydrated for 10 min at 110 °C and Microposit S1818 positive photoresist was spin coated (4000 rpm for 60 s) and annealed at 110 °C for 1 min. Metallic contacts were patterned through direct laser lithography by using the ML3Microwriter (from Durham Magneto Optics, Cambridge, UK). The photoresist was developed with a Microposit MF-319 developer. Then, 10 nm of chromium and 30 nm of gold were deposited by thermal evaporation. Samples were immersed in acetone for 4 h for photoresist liftoff and rinsed by sonication in acetone/isopropanol/distilled water baths. A double layer of S1818 was deposited for the photolithography of the PEDOT:PSS channel. After development, substrates were treated with air plasma (15 W for 4 min), and the PEDOT:PSS solution was spin coated at 3000 rpm for 10 s. The solution was made of 93.75% PEDOT:PSS (Clevios PH1000, provided by Heraeus Deutschland GmbH & Co., Leverkusen, Germany) with 5% ethylene glycol (EG) (Sigma-Aldrich, St. Louis, MO, USA), 1% 3-glycidoxypropyltrimethoxysilane (GOPS) (Sigma-Aldrich, St. Louis, MO, USA), and 0.25% 4-dodecylbenzenesulfonicacid (DBSA) (Sigma-Aldrich, St. Louis, MO, USA). This suspension was treated in an ultrasonic bath for 15 min and filtered using 1.2 μm cellulose acetate filters (Sartorius) before the deposition. The resulting film thickness was (100 ± 10) nm. The samples were subsequently baked at 120 °C for 1 h. Then, the photoresist was lifted off following the procedure reported above, and devices were submerged in distilled H_2_O for 1 h and dried with a nitrogen flux. The resulting OECTs presented both the channel (600 × 800 μm^2^) and gate (2100 × 2100 μm^2^) in PEDOT:PSS. Channel to gate distance was *d* = 2 mm. In the end, a polydimethylsiloxane (PDMS) transparent, cylindrical well, having an inner diameter and height of 12 and 8 mm, respectively, was bound to the device to realize the culture well. The final device layout presents two channels and one gate: the two channels account for nonhomogeneous cell layer formation. Presenting the device average values of two channels can strongly represent the whole cell culture behavior, avoiding misleading results occurring from cell nonhomogeneous distribution.

### Establishment of HCC Cell Line-PBMC Coculture

Huh7 and SNU449 cells were seeded on an OECT device with 20 thousand (20k) PBMCs in antibiotic-free culture medium. The same number of cells and PBMCs were cultured alone under the same conditions as the two control groups. 48 h later, epithelial cancer cells were detached by Trypsin/EDTA and cell cycle analyses and apoptosis quantification were performed by flow cytometry by using Cytoflex S (Beckman Coulter) daily checked with S calibration beads to keep the setting and histogram uniform during time.

### OECT Measurement Using the TECH–OECT Prototype

Experiments were performed using the integrated system, TECH–OECT, previously reported in our studies.^15,16^ Cells were seeded inside the cylindrical PDMS wells with or without lymphocytes to monitor the experiment remotely without any further operator interaction. TECH–OECT platforms allow one to carry out measurements inside the humidified incubator (constant temperature of 37 °C and a CO_2_ level of 5%) using a multiplexer system to sequentially sample 12 OECT channels (up to 6 different cell cultures). We measured the source–drain current by means of a Keysight B2912A Source Measure Unit (SMU), while biasing the channel with *V*_ds_ = −0.1 V and introducing a square wave potential on the gate electrode, from *V*_gs_(OFF) = 0.0 V to *V*_gs_(ON) = 0.3 V, with *t*_on_ = 0.5 s and *t*_off_ = 1.5 s. Keysight and the multiplexer were both controlled with customized PC software.

### OECT Data Analysis

A customized Matlab routine was used for data analysis: each single channel response to a pulse on the gate was isolated, normalized, and fitted with the biexponential curve: $I_{ds}=a\cdot \exp^{-t/\tau_{1}}+b\cdot \exp^{-t/\tau_{2}}+c$ , as reported in our previous work.^24^ As described by Salleo’s group,^25^ when τ_1_ is greater than τ_2_, τ_1_ represents the charging time of the PEDOT:PSS influenced by the ion-blocking properties of the cell layer, while τ_2_ relates to the charging time of the cell layer. Thus, τ_1_ will be extracted as device time response to a gate potential pulse, averaging its value over five pulses on the same channel and then normalizing it using the following equation: OECT Time Response (a.u.) = τ/τ_No_Cells_, with τ_No_Cells_ representing the response time of the device at the beginning of the experiment, immediately after cell seeding and thus without any cells adhered onto the devices.

### Electrochemical Impedance Measurements

To prove that Salleo’s group^25^ model represents and can be employed for our devices, impedance spectroscopy measurements were taken during a cell healthy growth onto the OECT using a two-electrode setup, namely, the transistor channel and gate. A potentiostat/galvanostat VERSASTAT3A from Photo Analytical s.r.l. has been used to apply a sinusoidal AC signal, 10 mV in amplitude, between the working electrode (short-circuited source and drain contact of the OECT) and the RE/CE (OECT gate electrode). The graphs in Figure S1 show that a R­(RC)­(RQ) correctly fits the impedance data acquired after 48 h of cells cultured onto the OECTs. The constant phase element (Q) has been introduced in place of the capacitance (C) to account for PEDOT:PSS capacitance, owing to its porous structure enabling charge accumulation.^26,27^ Moreover, impedance analysis taken after seeding (T2h), halfway (T23h), and at the end of the experiment (T47h) present little differences among the captured trends, demonstrating transistor benefit and amplification for cell health data extraction and evaluation (Figure S2).

### Cell Cycle and Cell Death Assay

Huh7 and SNU449 cells were collected, washed twice with PBS, and split for cell cycle and cell death analyses. For cell cycle evaluation, cells were fixed with 70% cold ethanol overnight at −20 °C. Fixed cells were resuspended in PBS with 10 μg/mL propidium iodide (Sigma-Aldrich) and 0.1 μg/μL RNase A (Sigma) and incubated for 20 min in the dark at room temperature. Cells were then centrifuged at room temperature at 1500 rpm for 5 min, resuspended in PBS, and analyzed on Cytoflex S for DNA content quantification. Apoptosis was revealed by Annexin V-FITC and propidium iodide (PI) staining (Bender Medsystems, Vienna, Austria) via FACS. When the experiments were repeated to check the reproducibility of the results, some optical images (red staining with Evos) were also acquired to show dead cells.

### Interleukin 6 Immunoassay

Human Interleukin-6 (IL6) was quantified in cell culture medium by ELISA (BMS213INST Invitrogen Kit) according to the manufacturer’s procedure. Absorbance was quantified by a Spark microplate reader.

### Cell Images

Morphological changes of cocultures compared to the controls were assessed by taking images with Evos. White-light interferometer images on cell cultures were obtained using the SmartWLI provided by GBP Metrology throughout the 50× objective lens for high resolution cell morphology. For this analysis, cells were fixed in ethanol 48 h postseeding.

### Statistical Analysis

Comparisons between groups were performed by unpaired Student’s *t*-tests. Reported *p* values were considered significant when they were lower than 0.05. Linear regression was used to explore the relationship between G1 phase of the cell cycle and OECT time response or between Annexin-V staining and OECT time response. Statistical calculations were executed using SPSS version 20.0 (SPSS inc): * *p* < 0.05; ** *p* < 0.01; **** *p* < 0.0001.

## Results

### PBMC Effects on Cancer Cells

Planar PEDOT:PSS-based OECTs were employed in this study, since we previously proved their high sensitivity to cell tissue disruption, once incubated with Sars-Cov-2 virus.^16^ The device’s schematic geometry and its design are shown in Figure 1A,B, while its fabrication procedure is reported in the Experimental Section. The device’s complete electrical characterization in 1× PBS is reported in Figure S3. The solid device fabrication and its reliable and reproducible response are demonstrated by the channel resistance and time response distributions, respectively, shown in Figure S4.

**Figure 1:**
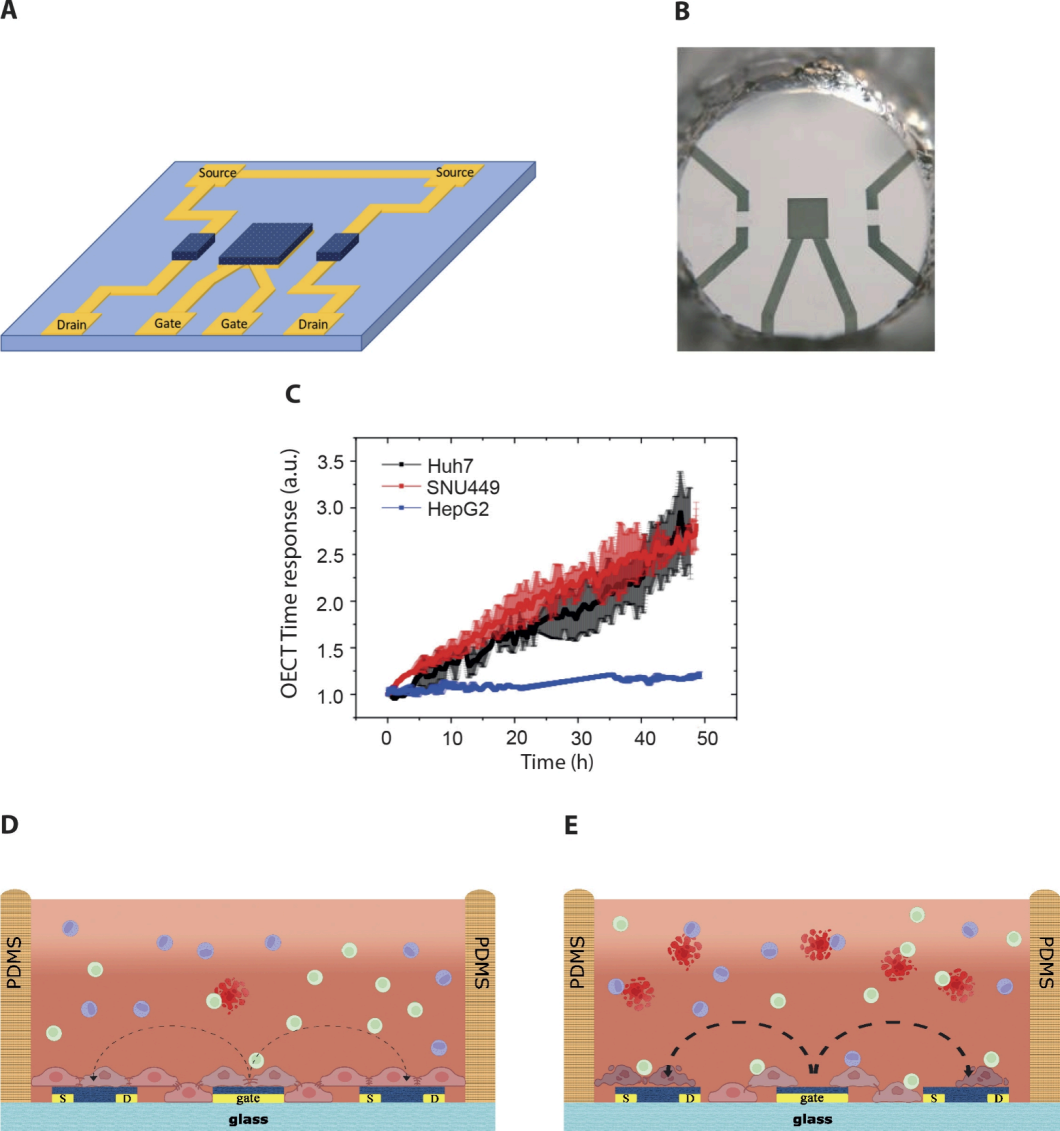
OECT scheme, device response to cell cultures, and assay cross sections and working principles. (A) OECT scheme with gold feedlines and PEDOT:PSS pattern: as reported in the Experimental Section, the configuration has two channels (600 × 800 μm^2^) and a gate (2100 × 2100 μm^2^) separated by a distance of *d* = 2 mm. (B) Picture of the single OECT unit having the PDMS well for the cell culture, which would be connected to the TECH–OECT platform for monitoring the device response. (C) Normalized OECT time response monitoring three healthy cell cultures (*n* = 3), namely, Huh7 (black line), SNU449 (red line), and HepG2 (blue line). The OECT time response is measured in arbitrary units (au) as it represents the following ratio: τ/τ_No_Cells_, as reported in the Experimental Section. (D, E) Schematic cross-section of the coculture assay behavior upon PBMC exposure taken from nonresponding (D) and responding (E) patients. The circular lines highlighted with a different pattern in D and in E show the ionic currents from the gate to the channel, which are slowed by the presence of healthy cell layers. Image created with BioRender.com.

HepG2 growing in multilayers gives an electrical signal like that of lymphocytes alone and is therefore not useful for the purposes of this study (Figure 1C and Figure S5A). Huh7 and SNU449 were interchangeably used as cellular models growing in monolayers (Figure 1C) with high and healthy adhesion (Figure S5B,C).

We proposed a coculture OECT assessment in which PEDOT:PSS-based OECTs monitor in real-time the adherent cell line growth cultured with 20k PBMCs (in suspension) isolated from patients. Depending on the effects of the PBMCs onto the adhered cell lines, we envision two different scenarios: we expect that PBMCs from nonresponding or immunocompromised patients would not affect the cells, thus having tight and healthy cell layers growing onto the OECTs, as schematically depicted in Figure 1D; conversely, active PBMCs from treatment-responding patients or healthy patients would target the cell layer, thus inducing cell stress and damage, as represented in the cross-section in Figure 1E.

Since only active lymphocytes can induce death or growth alterations in tumor cells cultured on an OECT, we hypothesized that they would measure lower parabolic responses over time due to stressed cell layers. In line, only PBMCs from healthy controls and patients responding to immunotherapy negatively influence the cell layer integrity as highlighted by a drop in the device time response (Figure 2A,B).

**Figure 2:**
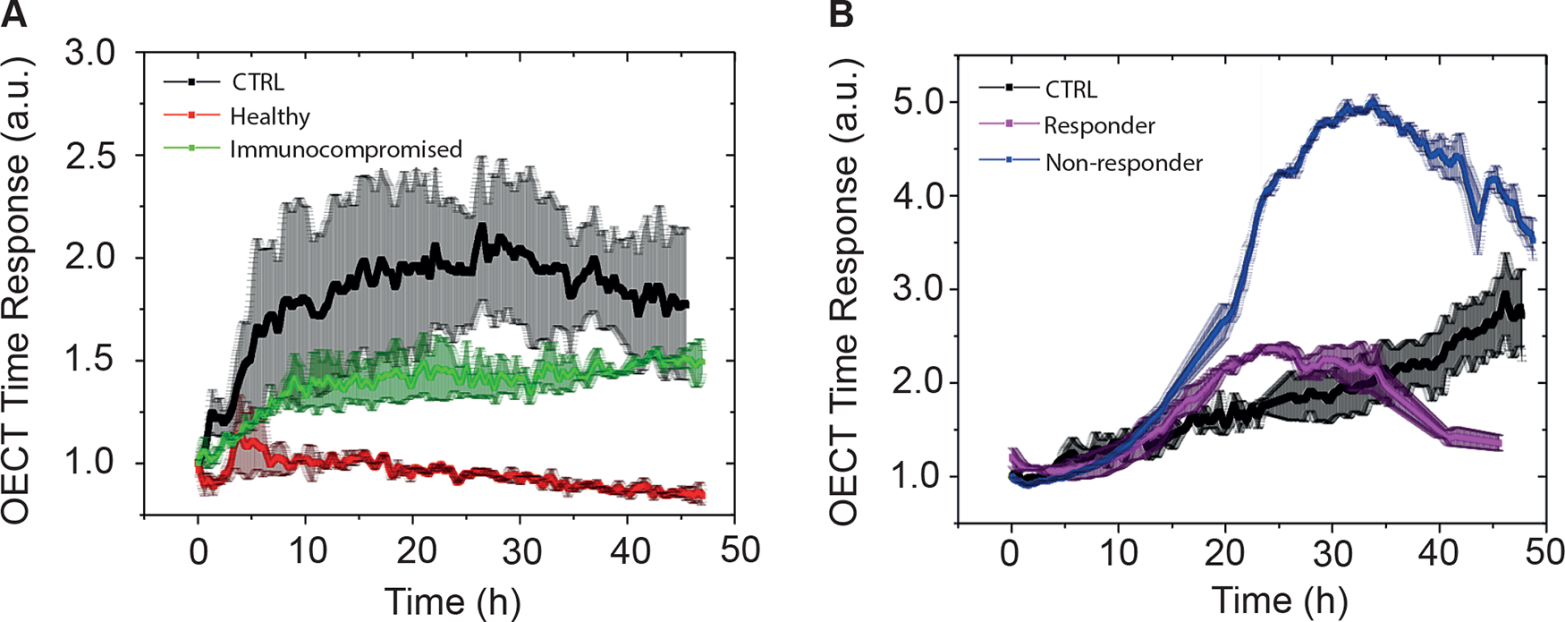
Average OECT normalized time response. Two different devices (*n* = 2) with standard deviations (shadows) monitoring cell behavior upon PBMC exposure with respect to the controls (black) are reported. (A) SNU449 cells seeded with PBMCs of healthy (red) or immunocompromised (green) patients. (B) Huh7 cells seeded with PBMCs of a responding (purple) and nonresponding (blue) patient. Graphs are representative of three independent experiments. The OECT time response is measured in arbitrary units (au) as it represents the following ratio: τ/τ_No_Cells_, as reported in the Experimental Section.

To ensure no significant biofouling effect during *in vitro* experiments (i.e., how the accumulation of proteins, cellular debris, or metabolites could affect cell health and device response), we cultured Huh7 in 50% and 100% medium retrieved from another 72 h similar cell culture experiment. As shown in Figure S6, cell health and transistor response are altered after 25 h in 100% recovered medium, whereas our experiments end within 48 h of cell seeding and the medium recovered after 72 h from a similarly grown cell culture does not alter the response for at least 25 h; any biofouling effect does not represent a limitation.

To validate our sensor response, optical images (in brightfield and using propidium staining) together with cytofluorimetric analysis were taken at the end of the experiments. Cytofluorimetric assay allowed us to understand whether a single administration of atezolizumab plays any effect on cancer cell proliferation (increased, reduced, or unaffected) with respect to PBMC activation (Figure 3). PBMCs isolated from responding patients to atezolizumab, as well as from healthy subjects, affect Huh7 and SNU449 proliferation by arresting cells in the G0/G1 phase (Figure 3A,B). Conversely, PBMCs isolated from immunocompromised or nonresponding patients had no effect on cancer cell cycles (Figure 3C,D).

**Figure 3:**
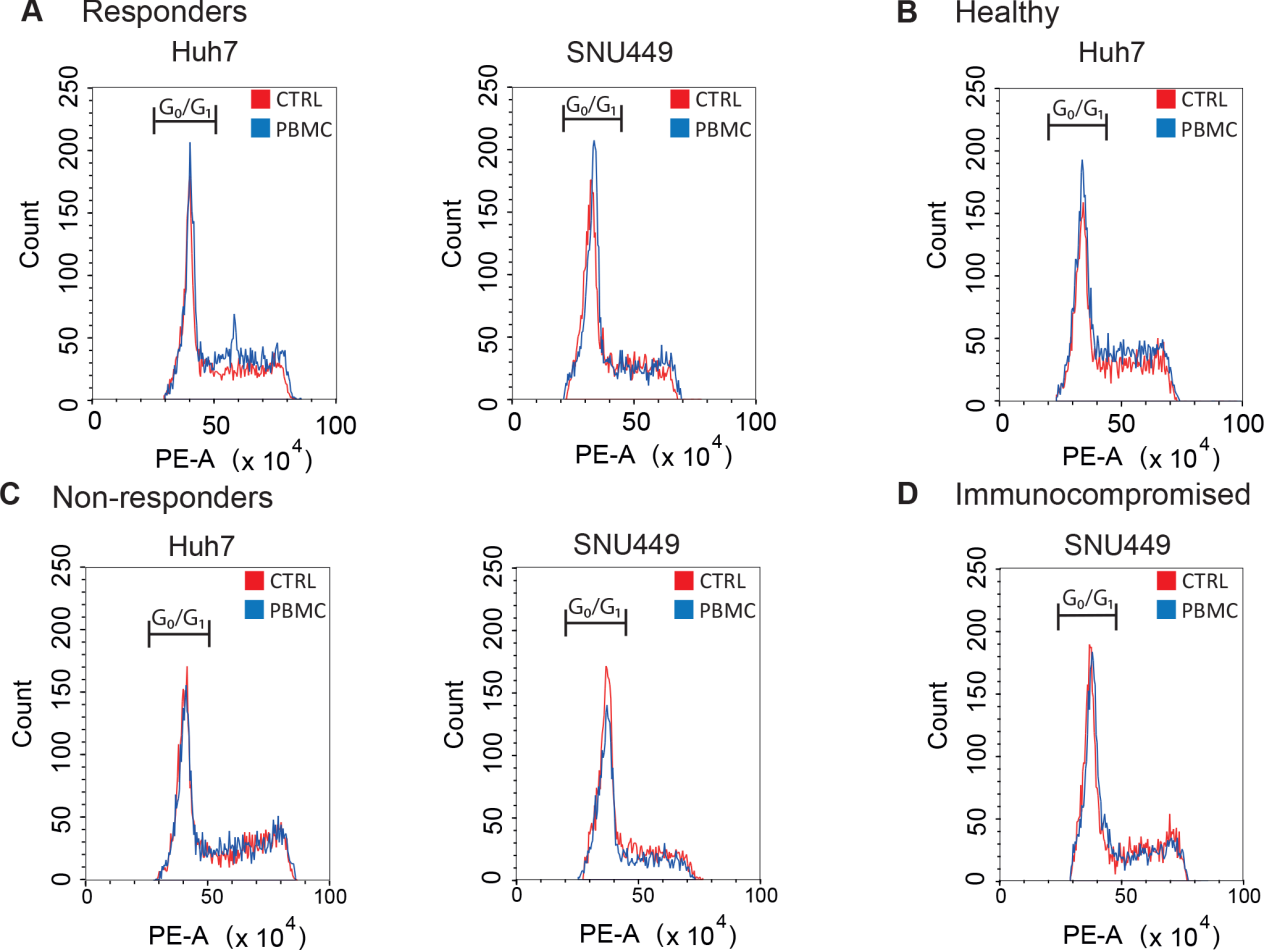
Effects of PBMCs on the proliferation and cell cycle of HCC-derived cell lines. Huh7 and SNU449 cells were harvested after being cultured with PBMC for 48 h, and propidium iodide staining was used to analyze cell cycle distribution. In the graphs, controls (CTRL) and cancer cells collected by cocultures with PBMC are shown overlapped to appreciate variations in cell cycle distribution. PBMCs from responders (A) and healthy subjects (B) produce an increase in G0/G1 phase, while in nonresponders (C) and immunocompromised (D), they produce a G0/G1 reduction.

Flow cytometric analysis was performed in at least three independent experiments resulting in similar results, thus ensuring the reliability of the findings. One experiment is shown as a representative.

The Annexin V cytofluorimetric analysis and the optical micrographs (red staining with Evos to show dead cells), acquired as end-point assays after 48 h, confirmed our electrical results, both reporting the increase in the cell death population upon exposure to PBMCs isolated from healthy and responding patients to atezolizumab–bevacizumab (Figure 4A,B). The same effects were not observed in nonresponders and in immunocompromised patients (Figure 4C,D).

**Figure 4:**
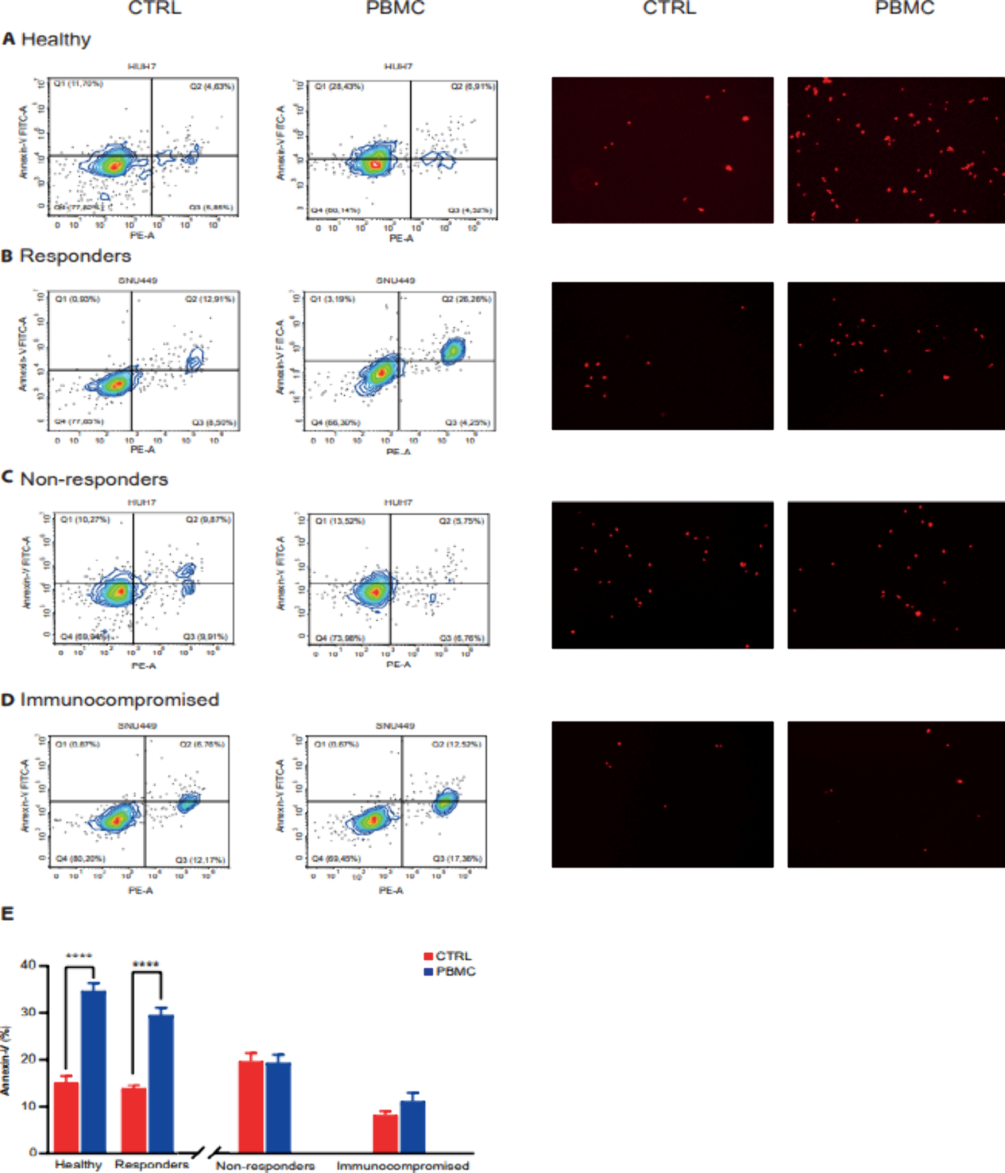
Cell death evaluation. After coculture with PBMC, Huh7 (A, C) or SNU449 (B, D) cells were labeled with annexin V-FITC and propidium iodide. The distribution pattern of alive and apoptotic cells was determined by FACS analysis. *X*-axis represents propidium staining (PE), and the *y*-axis represents FITC staining. Optical micrographs (right panels) after 48 h of coculture, using red staining dye for dead cells, were acquired with EVOS (Thermo Fisher Scientific, USA). Images are representative of at least three independent experiments. (E) Bar charts of Annexin V percentage values measured in PBMC-treated HCC cells vs control cells of three independent experiments. A significant increase in Annexin V measured in PBMC-treated cell lines was observed only in healthy and responding patients. ****p < 0.0001 by unpaired *t*-test.

An inverse correlation was found between the OECT time response and the G1 phase of the cell cycle, as well as between the response of the OECT and apoptosis (Figure S7), highlighting the relation between immune activation and the onset of the OECT readout. Looking for a marker associated with immune activation, we focused on IL6 whose secretion promotes tumorigenesis by influencing cancer hallmarks, such as apoptosis, proliferation, angiogenesis, metastasis, and cell metabolism.^28^ According to the OECT readout and flowcytometric results, IL6 levels analyzed in the coculture supernatant decreased in responders and increased or were not affected in nonresponders (Figure 5).

**Figure 5:**
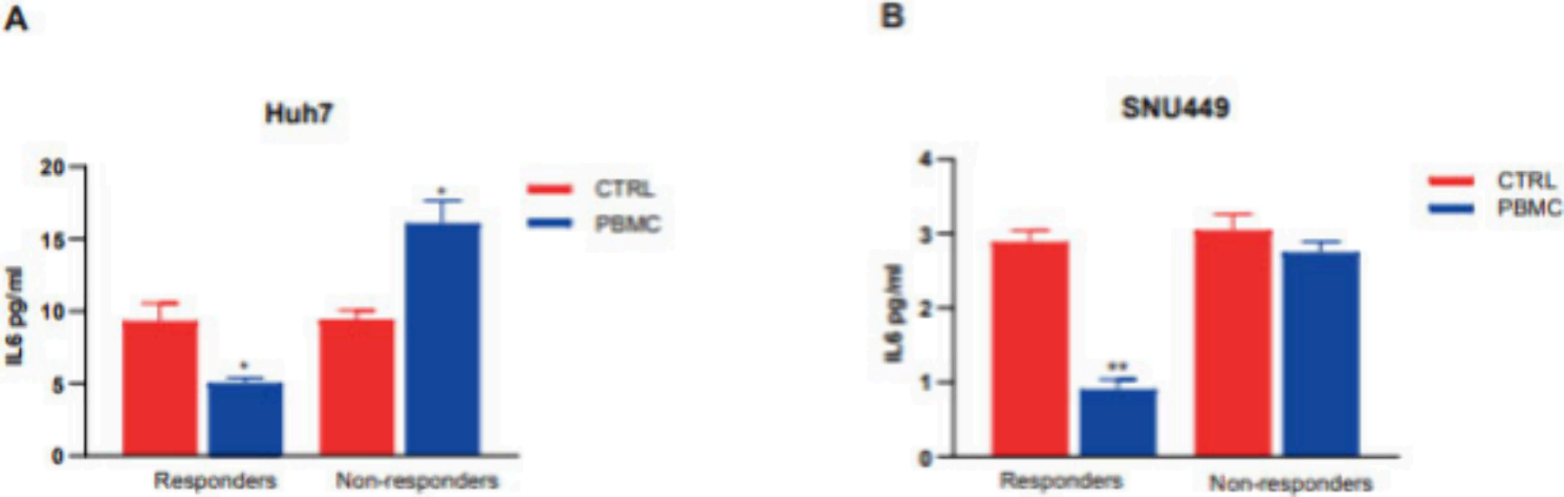
IL6 detection by ELISA for Huh7 (A) and SNU449 (B) cell lines. Data are expressed as means (±S.E.). *p* values were derived from a two-tailed student’s *t* test. *p < 0.05.

### Morphological Changes in Tumor Cells in Tested Cocultures

The cell culture morphology of Huh7 and SNU449 as well as their cocultures are presented in Figure 6. Significant changes in the structure of the tumor cells were observed only in coculture with PBMCs of healthy or responding patients, suggesting that they trigger a response when they come into contact with cancer cells. PBMCs induced the appearance of rounded epithelial cells within 24–48 h in both cell lines, a phenotypic feature typical of dying cells (Figure 6A–D). The remaining epithelial cells were polygonal in shape and had growth attached to the substrate. When analyzed by a white-light interferometer to evaluate the cell surface profile, PBMC activation reduced heights and boundaries among HCC cells compared with control cultures (Figure 6E). Cell boundaries are mediated by specialized structures called cell junctions that play a fundamental role in cancer biology. In this context, the functional role of junctions has been described in apoptosis with their disruption considered as an early event that initiates caspase activation and cell death.^29^

**Figure 6:**
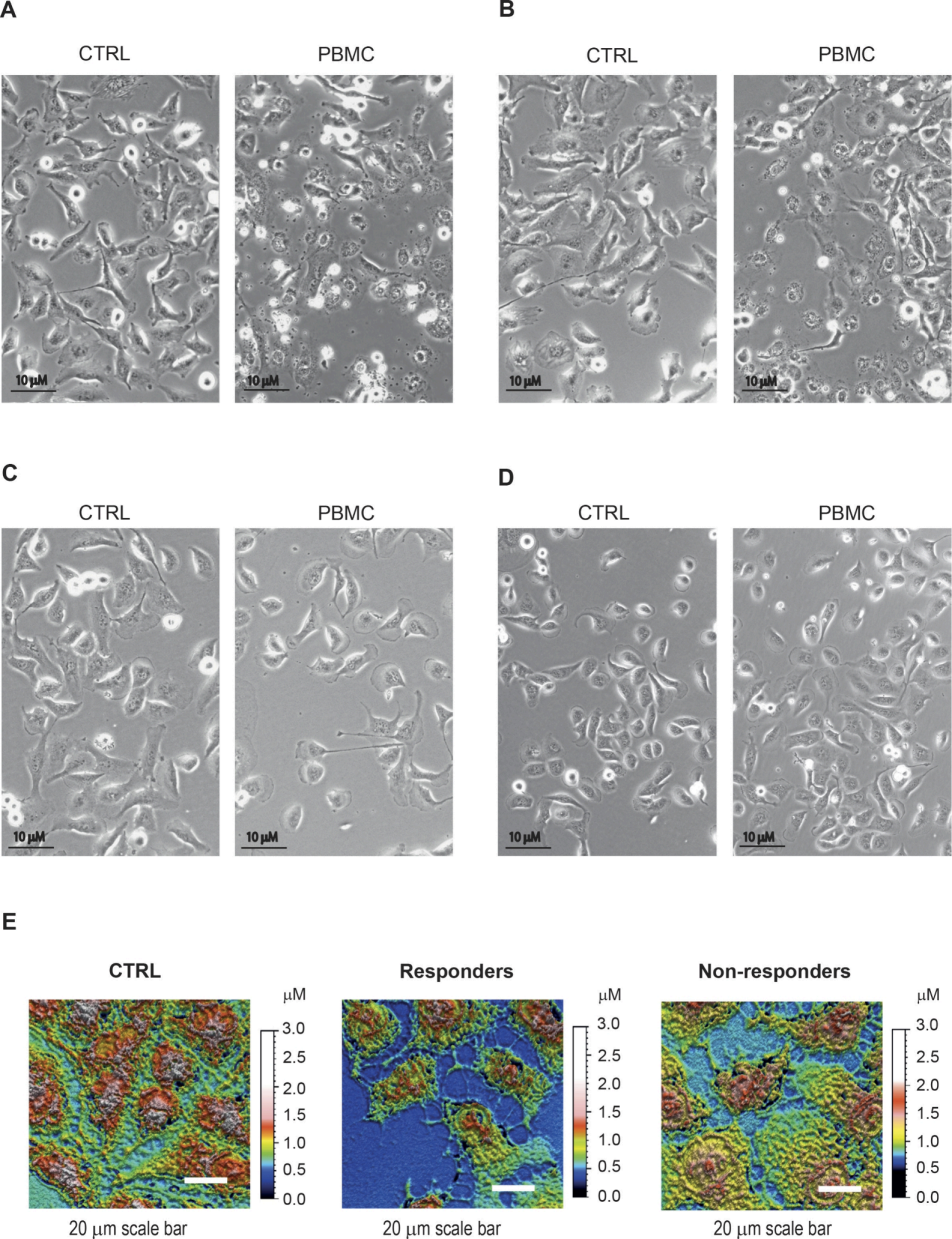
Morphological investigation through optical and interferometer imaging. Bright field microscope images show SNU449 cell stress induced by PBMC from a healthy control and a patient responding to atezolizumab–bevacizumab (A, B) and from an immunocompromised or nonresponding patient (C, D). Black scale bars in the micrographs measure 10 μm. (E) White-light interferometer images of cells cultured 48 h onto the OECT with or without 20k PBMCs in a responding and in a nonresponding patient show a decrease in cell–cell junctions in responders. White scale bars in the micrographs measure 20 μm.

### OECT Screening of Missed Outcomes

By using a cytofluorimetric test, we observed that the majority of HCC patients responding to atezolizumab–bevacizumab are characterized by an increasing fold change (FC) of CD8+PD1+ at 3 weeks (T1) compared to baseline (T0) in PBMCs, suggesting this dynamic change is a potential biomarker. Conversely, most patients with disease progression showed a negative FC due to a decrease in CD8+PD1+ lymphocytes.^22^ However, the CD8/PD1 fold change fails to correctly distinguish responders and nonresponders in about 15% of cases. OECT screening correctly identified all, but one patient missed early CD8+PD1+ changes. Examples of a nonresponder and a responder missed by CD8/PD1 FC are reported in Figure 7A,B, respectively, providing a valuable approach to complement clinical and imaging evaluations in the very early phases of atezolizumab–bevacizumab treatment of HCC.

**Figure 7:**
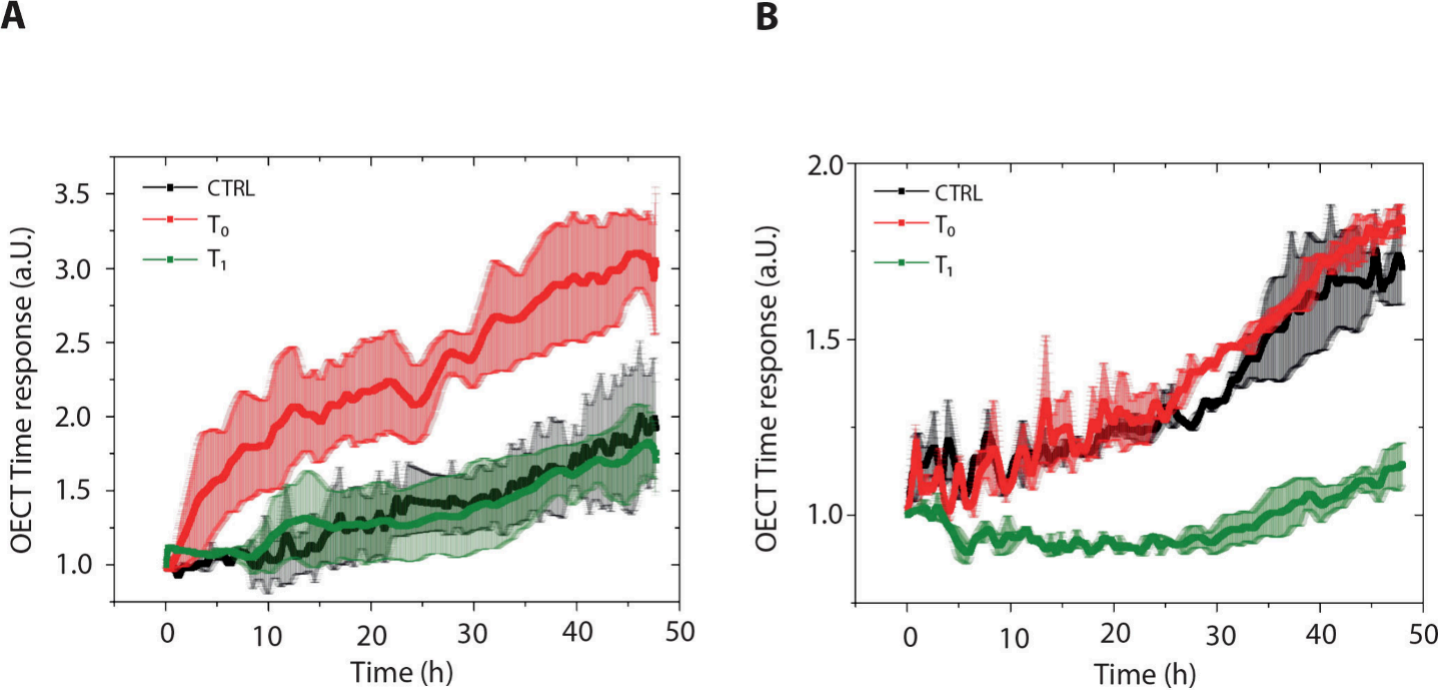
Screening of missed outcomes using OECTs. (A) Average OECT normalized time response over two different devices (*n* = 2) with standard deviations (shadows) investigating the PBMC effects on SNU449 from baseline (T0 in red) and 3 weeks post-treatment (T1 in green) blood sampling of a missed, nonresponding patient, compared to the control (black). This patient showed a positive CD8+PD1+ FC (T1/T0) instead of a negative one. (B) Average OECT normalized time response over two different devices (*n* = 2) with standard deviations (shadows) investigating PBMC effects on Huh7 before (T0 in red) and post-treatment (T1 in green) blood sampling of a missed, responding patient, compared to the control (black). This patient showed a negative CD8+PD1 FC (T1/T0) instead of a positive one. The OECT time response is measured in arbitrary units (a.u.) as it represents the following ratio: τ/τ_No_Cells_, as reported in the Experimental Section.

## Conclusion

Immune checkpoint inhibitors (ICIs), such as programmed death ligand 1 (PD-L1) and programmed cell death protein 1 (PD-1), have revolutionized HCC treatment by enabling a durable response and extending survival.^30^ Despite the high success, approximately 30% of patients remain unresponsive to ICI therapy. Such a rate underlines the critical need for predictive tests that help to optimize treatment strategies, since more options are available in the front line, ultimately improving clinical outcome.

We demonstrated, for the first time, that organic electrochemical transistors represent a reliable approach for monitoring the early response to atezolizumab–bevacizumab treatment in advanced hepatocellular carcinoma with broad prospects for clinical translation. OECTs monitoring PBMCs’ activation during immunotherapy provided advantages in terms of (i) simple assay procedure and protocol and (ii) quantitative objective analysis, owing to the automatized TECH–OECT prototype. Furthermore, allowing for a continuous sampling of the cell culture’s health status, the assay could be continuously and remotely supervised, reducing experienced-operator workloads and time-consuming subjective evaluations. Most importantly, our work showed that this in vitro technology allows correct categorization of patients missed by other tests (either responders or nonresponders), envisioning its coupling with laboratory standards for enhancing the outcome robustness.

However, it must be noted that large amounts of lymphocytes suspended in the culture or leaky barrier cell lines (i.e., HepG2) may be challenging for OECT monitoring: the former can introduce unwanted drift signals due to impedance footprint; the latter may have cell tissue resistance below the OECT limit of detection. Therefore, we cannot say with confidence that HepG2 cells do not respond to PBMCs. To reduce lymphocyte effect, wider OECT geometries should be employed, while smaller ones have to be designed for monitoring leaky-barrier cells: consequently, a trade-off must be found, solving/targeting the specific issue by customizing the OECT channel and gate dimension, as explained in a previous work by Fariat et al.^31^ and demonstrated for cells having different epithelial resistances by our group.^32^

Currently, the TECH–OECT platform allows up to six different conditions to be analyzed in real time, but device scalability and polymer ease of processability would allow industrial fabrication for multiple analysis and worldwide spreading of the assays with higher automated reproducibility. We are currently working on the prototype platform scale-up, aiming at high-throughput and reliable analysis of up to 48 OECTs. This technology may find application in monitoring the response to immunotherapy in various solid human cancers with the advantages of cost effectiveness, portability, and ease of use.

## Supplementary Material



## Data Availability

The data will be provided by the authors upon reasonable request.

## Notes

Figure S1: Impedance analysis for OECT equivalent circuit; Figure S2: Impedance analysis monitoring cell culture growing onto OECTs; Figure S3: OECT characterization; Figure S4: Batch uniformity and reproducibility; Figure S5: OECT response to PBMC and optical view; Figure S6: Biofouling effect of cell media; Figure S7: Statistical correlation between cell phase/death and OECT response (DOCX)
