# Supplementary material for: Organic Electrochemical Transistors for Real-Time Detection of Immune System Early Activation during Atezolizumab-Bevacizumab Treatment in Hepatocellular Carcinoma
Source: ACS Appl Mater Interfaces. 2026 Apr 29;18(18):25892–901. doi: 10.1021/acsami.6c03306 (PMC13181722; doi:10.1021/acsami.6c03306)
Supplement: Supplementary file 1 [file am6c03306_si_001.docx]

**Supporting Information**

**Organic electrochemical transistors for real-time detection of immune system early activation during atezolizumab- bevacizumab treatment in hepatocellular carcinoma**

Francesco Decataldo,^*†1^, Andrea Arleo^†1^, Annapaola Montagner,^1^ Giorgio Cortelli,^2^ Cristina S. Cioroianu,^1^ Fabio Piscaglia,^1,3^ Beatrice Fraboni,^2^ Laura Gramantieri,^3^ Catia Giovannini^*1,3^

^1^ Department of Medical and Surgical Sciences, Alma Mater Studiorum - University of Bologna, 40138 Bologna, Italy.

^2^ Department of Physics and Astronomy, Alma Mater Studiorum - University of Bologna, 40127 Bologna, Italy.

^3^ Division of Internal Medicine, Hepatobiliary and Immunoallergic Diseases, IRCCS Azienda Ospedaliero-Universitaria of Bologna, 40138 Bologna, Italy.

*Corresponding authors: [francesco.decataldo2@unibo.it](mailto:francesco.decataldo2@unibo.it); [catia.giovannini4@unibo.it](mailto:catia.giovannini4@unibo.it)

^†^These authors contributed equally.

**Impedance analysis of the cell culture growing onto OECTs**

To demonstrate the suitability of Salleo’s group model and the correctness of the bi-exponential fitting for extracting OECT time response as reliable and quantitative parameter, we analyzed the two-electrode impedance of the *in vitro* system: cells were cultured onto the OECT and impedance measurements have been carried out daily, using OECT gate and channel as electrodes.


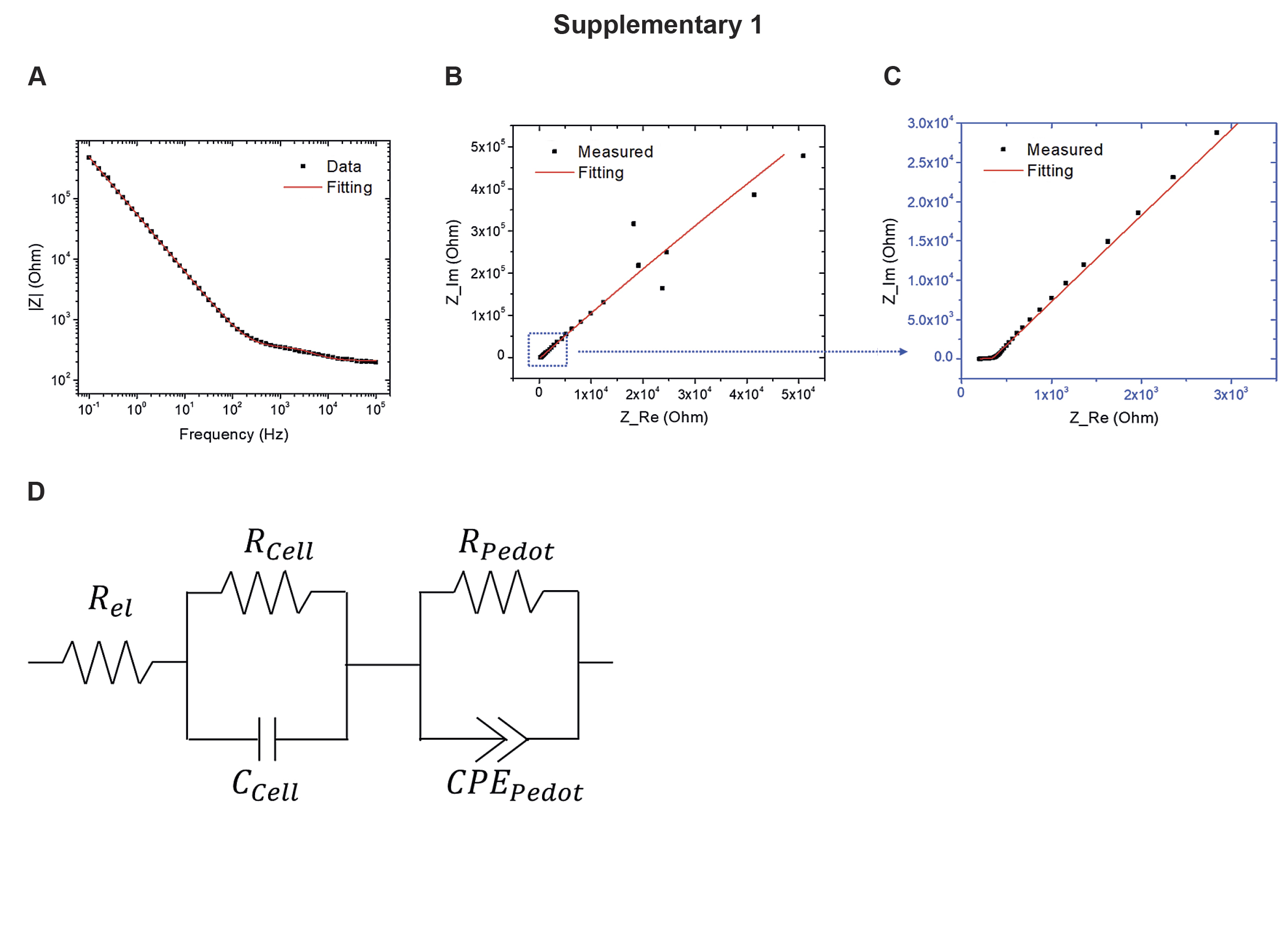


**Figure S1:** Bode (A) and Nyquist (B and C) plot reporting the electrochemical impedance analysis of the OECT device after 47h from cell seeding, together with equivalent circuit fitting (D), accounting for polymer and cell layer electrochemical response.

Evaluating impedance analysis taken after seeding (T2h), halfway (T23h), and at the end of the experiment (T47h), negligible differences have been observed among the impedance modulus and the Nyquist plots, as can be seen in SI Figure 2. This is coherent with the well-known transistor amplification, which boosts the system's sensitivity towards cell impedance.


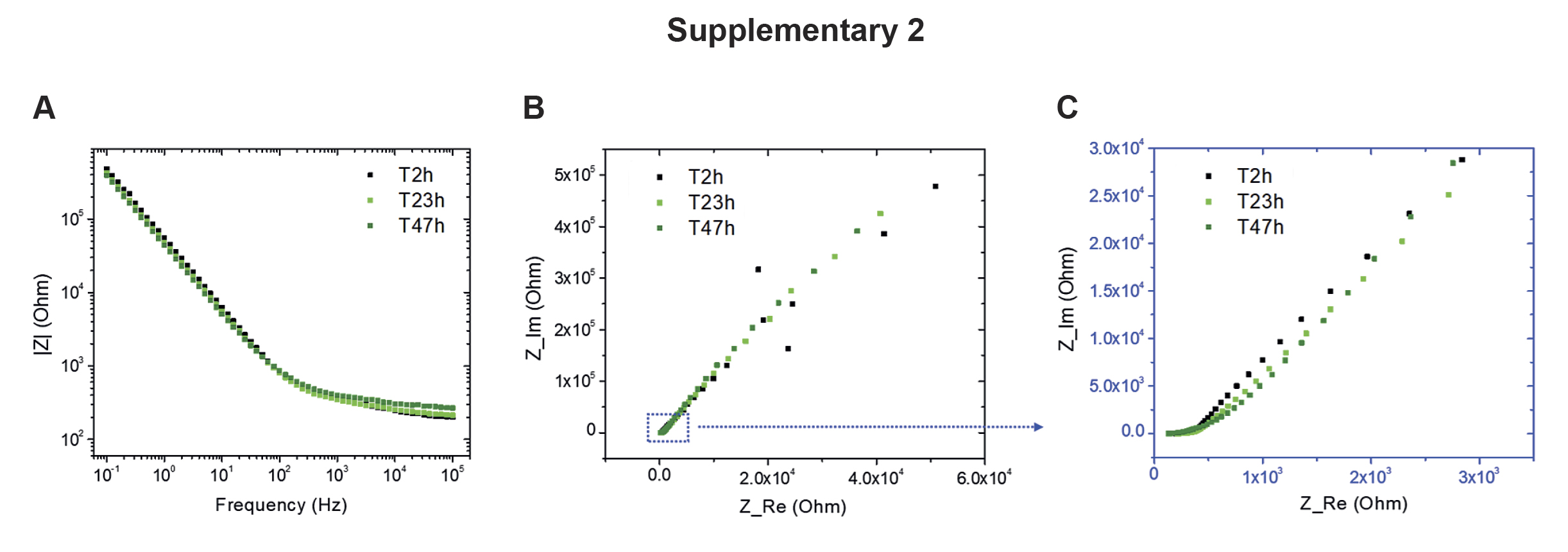


**Figure S2:** Bode (A) and Nyquist (B and C) plot reporting the electrochemical impedance analysis of the OECT device after seeding (black dots, T2h), halfway (light green dots, T23h), and at the end of the experiment (dark green dots, T47h), reporting negligible differences for growing and spreading cell layers.

**OECT device characterization**

SI Figure 3A, 3B, and 3C report the device transfer, output, and transient drain current response to square gate voltage pulses, respectively, providing the full OECT characterization. The device shows typical current modulation upon gate voltage variations.

SI Figure 3D reports the device gate current upon gate voltage variation extracted during a transcharacteristic curve. The low I_G_ signal, below 2μA in the whole V_G_ = [-0.5 ÷ 0.5V] range, confirms negligible leakage of the transistors. Thus, no shielding has been patterned for the narrow gold feedlines, avoiding potential non-compatible materials negatively affecting the cell culture (and, consequently, the assay).


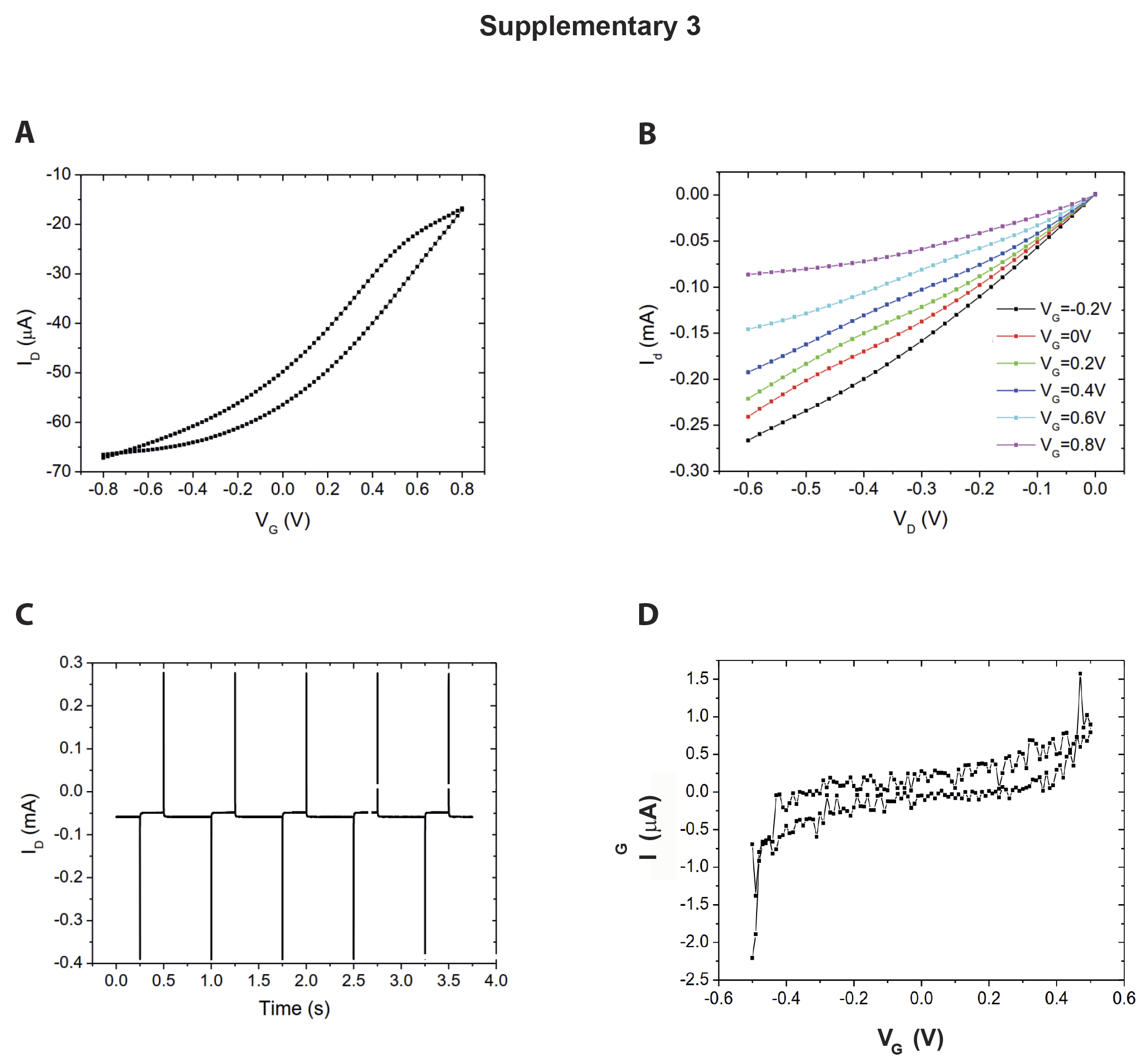


**Figure S3:** OECT transcharacteristic (A), output (B), and drain current transient response upon square voltage potential on the gate (C) obtained using PSB 1X as electrolyte. The transfer was acquired with V_d_ = -0.1V, Scan Rate=40mV/s. The pulsed measurement is acquired with V_d_ = -0.1V, V_gs_(ON) = 0.3V, and V_g_(OFF) = 0.0V. D) Gate (leakage) current as a function of gate voltage extracted from OECT transcharacteristic measurement in RPIM culture medium.

**Reproducibility and batch uniformity**

To evaluate fabrication batch uniformity and the reproducibility of the device, the channel resistance and the device time response at the beginning of *in-vitro* experiments (before cell adherence) were analyzed. As visible below in SI Figure 4, despite few outliners, the device resistances and time responses are centered around 600 Ohm and 1.5ms, respectively. The lithography-based fabrication proved to be effective and reliable, and further study can investigate its scalability for high-throughput assays.

**Figure S4:** OECT channel resistance (A) and time response (B) distribution for different fabrication batches. Bin sizes are 100 Ohm and 0.2 ms, respectively.

**PBMC response and cell homogeneity distribution**

To monitor adherent cell response to PBMC, it was mandatory to understand whether PBMC were able to affect OECT behavior. OECTs having only PBMC introduced into the culture medium presented steady time response (eventually fluctuating) which strongly differ from culture growth increasing trends, thus not affecting the in-vitro assessment (SI Figure 5). It must be noted that the HepG2 cell line gave a low increasing signal which overlaps with the PBMC one, thus not being suitable for these tests. Finally, Huh7 homogeneous growth onto the device surface optical micrographs are reported in SI Figure 5B-C, showing high and healthy cell spreading and adhesion both around and onto the PEDOT:PSS channel.


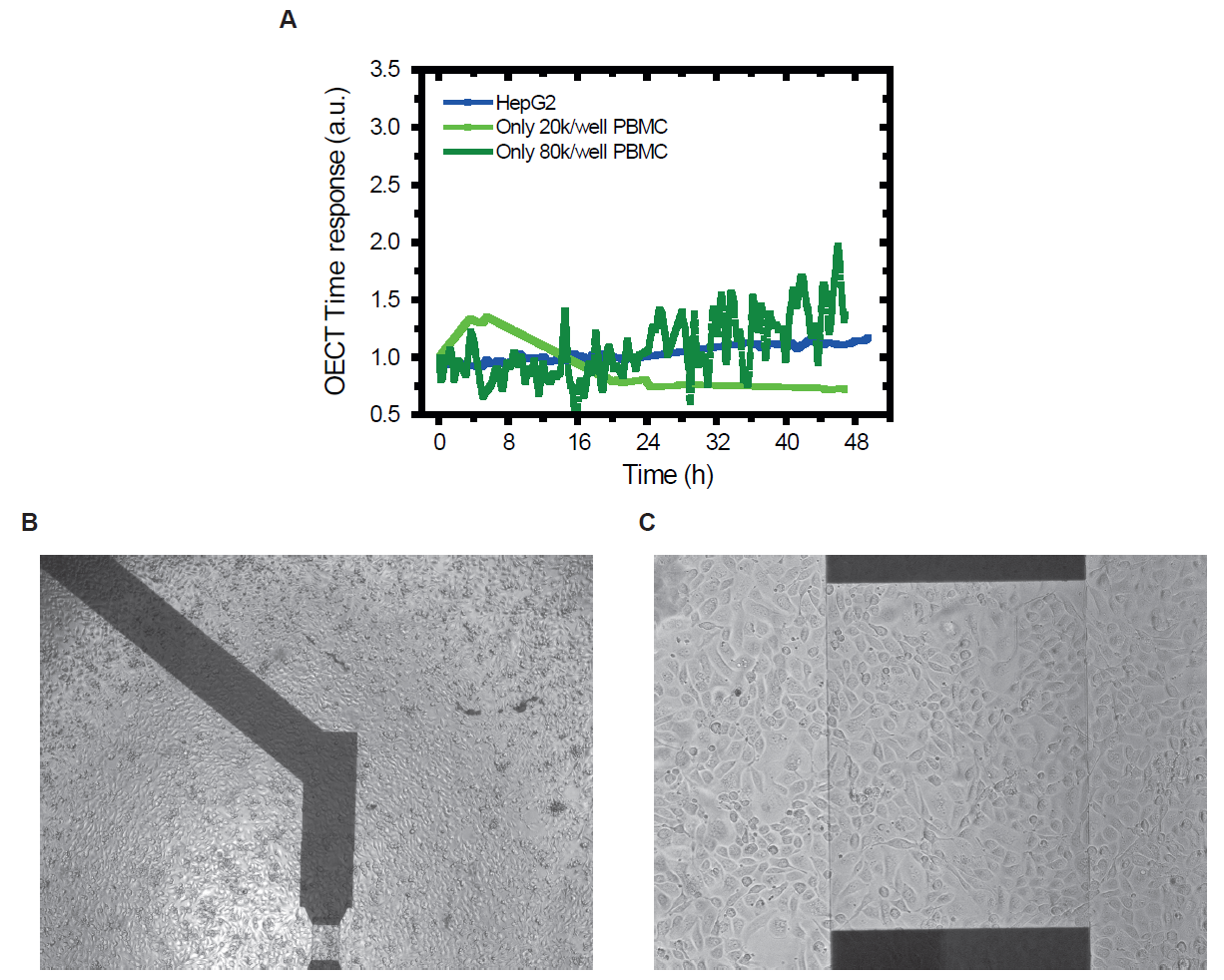


**Figure S5:** A) OECT time response for HepG2 (blue line), 20,000 PBMC/well (light green line) and 80,000 PBMC/well (dark green light). Optical micrographs showing the homogeneous cell spreading and growth around (B) and onto (C) the channel of a device.

**Assessment of biofouling effect**

To evaluate the biofouling effect of proteins, cell debris or metabolites produced during the cell culture over long *in-vitro* experiment onto OECTs, we monitored Huh7 growth onto the devices cultured in 50% (A) and 100%(B) of medium recovered from Huh7 cultured for 72h.


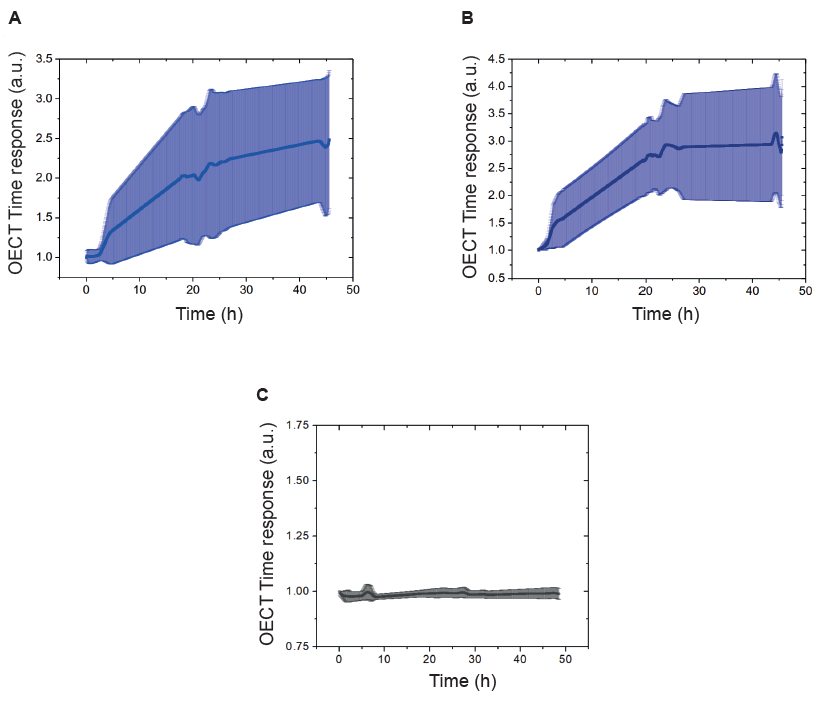


**Figure S6:** Average OECT normalized time response over three different devices (n=3) with standard deviations (shadows) for Huh7 cultured in 50% (A) and 100% (B) of medium, previously employed for standard growth of Huh7 for 72h, and an OECT without any cell seeded on top (C).

**Statistical correlation between the G1 cell cycle phase/cell death and OECTs’ time response in Huh7 and SNU449 cell lines**


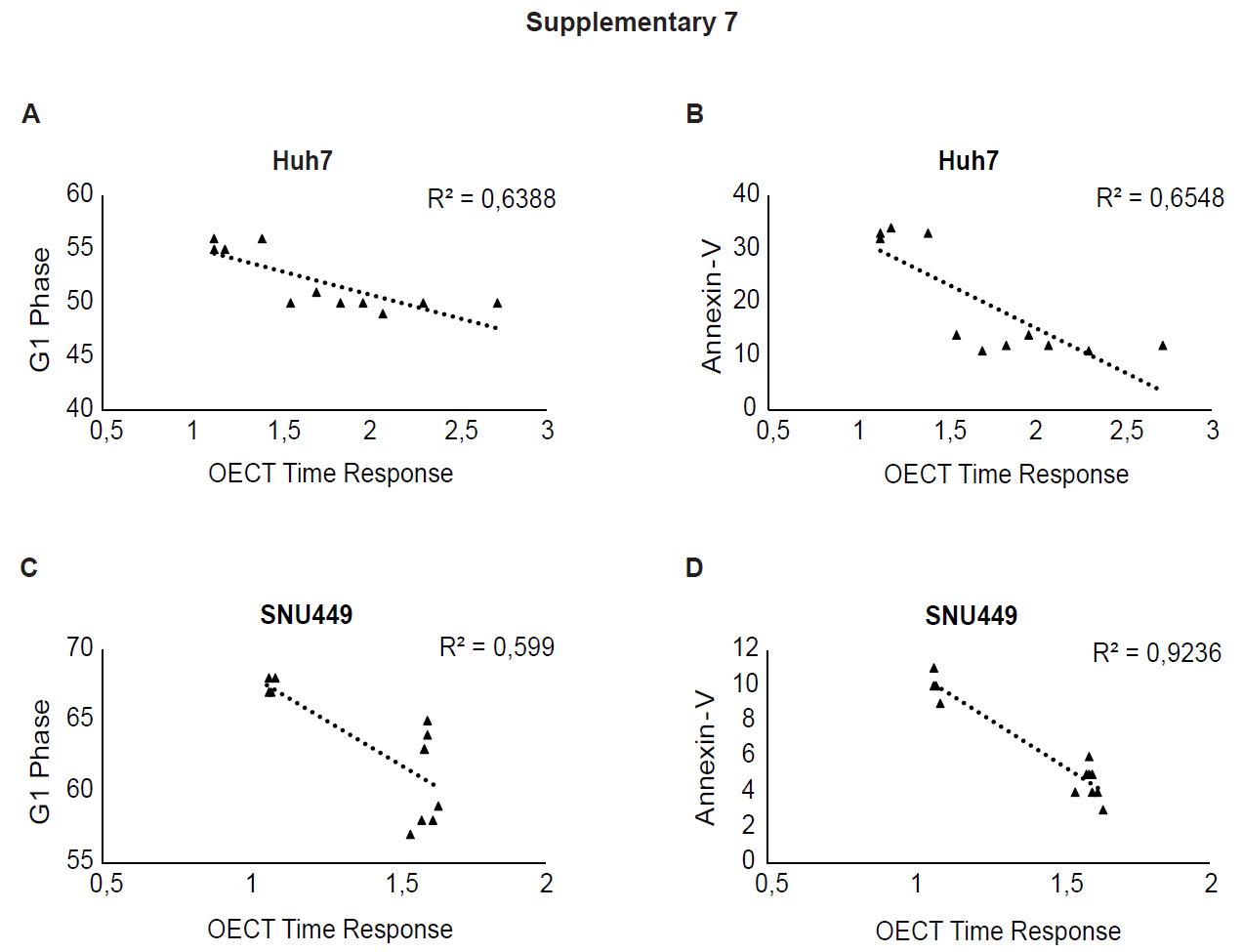


**Figure S7:** Regression analysis showing a negative correlation between the G1 phase of the cell cycle (A, C) and cell death evaluated by Annexin-V (B, D) with OECT Time response in Huh7 and in SNU449 cell lines.
